# Development and evaluation of a clinical decision support system for early diagnosis of acute appendicitis

**DOI:** 10.1038/s41598-023-46721-9

**Published:** 2023-11-11

**Authors:** Leila Shahmoradi, Reza Safdari, Mir Mikail Mirhosseini, Sorayya Rezayi, Mojtaba Javaherzadeh

**Affiliations:** 1 Health Information Management and Medical Informatics Department, School of Allied Medical Sciences, Tehran University of Medical Sciences, Tehran, Iran; 2 General Surgery and Thoracic Surgery, Modarres Hospital, Shahid Beheshti University of Medical Sciences, Tehran, Iran

**Keywords:** Computational biology and bioinformatics, Diseases, Health care

## Abstract

The most frequent reason for individuals experiencing abdominal discomfort to be referred to emergency departments of hospitals is acute appendicitis, and the most frequent emergency surgery performed is an appendectomy. The purpose of this study was to design and develop an intelligent clinical decision support system for the timely and accurate diagnosis of acute appendicitis. The number of participants which is equal to 181 was chosen as the sample size for developing and evaluating neural networks. The information was gathered from the medical files of patients who underwent appendicectomies at Shahid Modarres Hospital as well as from the findings of their appendix samples' pathological tests. The diagnostic outcomes were then ascertained by the development and comparison of a Multilayer Perceptron network (MLP) and a Support Vector Machine (SVM) system in the MATLAB environment. The SVM algorithm functioned as the central processing unit in the Clinical Decision Support System (CDSS) that was built. The intelligent appendicitis diagnostic system was subsequently developed utilizing the Java programming language. Technical evaluation and system usability testing were both done as part of the software evaluation process. Comparing the output of the optimized artificial neural network of the SVM with the pathology result showed that the network's sensitivity, specificity, and accuracy were 91.7%, 96.2%, and 95%, respectively, in diagnosing acute appendicitis. Based on the existing standards and the opinions of general surgeons, and also comparing the results with the diagnostic accuracy of general surgeons, findings indicated the proper functioning of the network for the diagnosis of acute appendicitis. The use of this system in medical centers is useful for purposes such as timely diagnosis and prevention of negative appendectomy, reducing patient hospital stays and treatment costs, and improving the patient referral system.

## Introduction

The appendix is a worm-shaped appendage that is located at the beginning of the large intestine called the cecum^1^. The most common factors that block the appendix include fecaliths, enlarged lymph nodes, and concentrated barium used in radiography and intestinal worms. Acute appendicitis occurs when white blood cells build up in the thickness of the appendix wall and cause it to be filled with pus. In acute gangrenous appendicitis, high inflammation disrupts blood flow to the appendix, increases the inflammatory process, and destroys tissue by blocking the blood supply^2^. According to statistics, 7% of people will experience acute appendicitis at some point in their lives, most frequently between the ages of 10 and 30. The lifetime risk of acute appendicitis is higher in men than women and also higher in the second decade of life^3^.

The inflammatory process that occurs in the appendix manifests itself as diffuse abdominal pain that briefly concentrates in the lower right abdomen. In addition, following inflammatory responses and abdominal pain, increased white blood cells and fever are the primary symptoms of acute appendicitis^4,5^. Continued mucous discharge rapidly leads to the dilation of appendix duct which leads to inflammation, gangrene, and perforation^6^.

The main biochemical tests used to diagnose appendicitis are those that determine inflammation. By alone, this method indicated a negative appendectomy rate of 12.3–19%. Even with the significant advancements in contemporary radiography, imaging, and diagnostic laboratory testing, accurately diagnosing acute appendicitis is still difficult^7^. Among the imaging modalities, ultrasound is a non-invasive, safe, cheap, and available approach that exists in most medical centers and is used as the selective imaging method in the diagnosis of appendicitis, and it also has a relatively optimal accuracy (87–96%)^8,9^. Furthermore, Computed Tomography (CT) scan is an accurate way to diagnose other inflammatory processes that appear in the form of acute appendicitis. Numerous studies have shown that the use of CT scans in diagnosing acute appendicitis has reduced the rate of negative appendectomy^10,11^. Although CT scans have offered significant advantages in diagnosis, they also have major disadvantages. This method exposes the patient to radioactive radiation, the cost of performing this technique is high, it cannot be used for pregnant women, and an allergy to the contrast material, either intravenous or oral, exists in some people. This method is not always available, and it takes more time to perform than the other imaging approaches^12^. These two imaging methods are widely used to diagnose acute appendicitis, however, the rate of misdiagnosis of acute appendicitis is still seen^13,14^. Additionally, in all health centers, especially in local or district health centers and un-advanced clinics, there may not be a physician specializing in surgery, and radiography and ultrasound equipment may not be available. Even if radiography and ultrasound equipment is available, the person interpreting the results may not have enough experience. Considering the difficulty of diagnosing acute appendicitis, it may be difficult to diagnose the disease in the mentioned centers or to plan for referrals to more advanced medical centers^12,15^.

Several scoring systems, such as Alvarado's scoring system and Ripasa's scoring system, have been developed to assist in the diagnosis process and to more accurately identify the subset of patients who would necessitate additional investigation, monitoring, or immediate surgical intervention^16,17^.

Notably, since these methods require specialized equipment and experienced radiologists, machine learning, a different method for diagnosing the disease, has been used. Data mining uses techniques crafted by machine learning and is a methodology to discover hidden patterns from large datasets using statistical approaches. Therefore, due to the challenges of early detection of appendicitis, artificial intelligence and data mining techniques can be considered to help with the diagnosis. In recent decades, artificial intelligence as a branch of advanced computer-based medicine has grown significantly. Artificial intelligence helps to create and apply medical knowledge and is widely used in the production and use of alerts and reminders^18^. In addition, the use of machine learning techniques to diagnose primary diseases has attracted the attention of researchers around the world^19^.

Clinical Decision Support Systems (CDSSs) are used to solve problems in the field of artificial intelligence that arise during the provision of health care by analyzing patient-specific data and deciding on the best solution among the various options^20^. In a research, appendicitis data was used to test the efficacy of logistic regression classifiers and fuzzy inference systems^21^. The study used the R programme to analyse samples chosen from the Knowledge Extraction based on Evolutionary Learning (KEEL) database, and the accuracy values of the logistic regression and fuzzy inference system results were high. Gender, abdominal pain, age characteristics, leukocyte, platelet, lymphocyte, neutrophil, and C-Reactive Protein (CRP) values were used in the data processing procedure along with the clustering method. Four ideal clusters were found to be present in the investigation. In order to create the best decision support system possible, this clustering study took into account the opinions of physicians^22^.

In another study, an artificial intelligence-based system has been developed to diagnose acute appendicitis with Support Vector machines (SVM). The included features were recruited from the records of 760 patients. The performance of this program has been compared with the Alvarado Clinical Scoring System (ACSS) and MultiLayer Neural Networks (MLNN). This program outperforms ACSS and MLNN with an accuracy higher than 99%. Machine learning prediction methods, such as the Radial Basis Function (RBF), Bayesian Networks (BN), and Multilayer Perceptron (MLP), have been developed to assist physicians in making the best choices and receiving precise information regarding whether to operate on patients with appendicitis. With this study, 95% of the diagnoses were accurate^23^. In a study conducted by researchers in 2021^24^, to analyse blood samples from children and adolescents with appendicitis, a detection system based on machine learning approaches (logistic regression, random forests, and gradient boosting machines) was created. There are 430 cases in the study's database between the ages of 0 and 18. The study's most dependable accuracy result, when employing random forest classification, is 94%.

According to the increase in the use of machine learning and data mining techniques in the field of helping to accurately diagnose diseases, our main contribution is the mining of clinical data of patients, physical examinations, and laboratory parameters using machine learning techniques to help accurately and timely diagnosis of acute appendicitis. There is a trend towards non-surgical management of appendicitis, leading to an increased need for correct preoperative diagnosis and classification. For medical purposes, machine learning-based techniques can be learned and widely used to aid in diagnosis. Data mining techniques are reliable and effective methods that provide high classification accuracy. We have evaluated whether we can help diagnose acute appendicitis and distinguish uncomplicated from complicated cases using machine learning algorithms. Hence, the main aim of this study was to design and evaluate a clinical decision support system. This clinical decision support system was developed to help diagnose acute appendicitis, and it has a simple user interface and a low implementation cost compared to other diagnostic approaches.

The following is the remainder of the article's structure. The materials and methods are given in the next section. The experimental results of the techniques are provided in "Findings" section. The conclusions and upcoming efforts for enhancements are covered in the final part.

## Materials and methods

This is a descriptive-developmental study that was conducted in three phases. In the first phase, we distinguished the features required to develop a CDSS. In the second phase, the CDSS was developed; in the third phase, the designed CDSS was evaluated.

### Determination and confirmation of main diagnostic features

In the descriptive phase, information was obtained by referring to the existing guidelines and standard instructions in the field of diagnosis and treatment of acute appendicitis. Diagnostic features were extracted from the literature, and clinical guidelines were surveyed by the surgeons for confirmation and documentation. The reviewed literature included Schwartz's Principles of Surgery, the book of Surgical Emergencies in Clinical Practice, Maingot’s Book of Abdominal Operations, and hospital resources available in hospital libraries. Based on this literature, some scoring approaches have been recommended. These methods include Ripasa’s scoring system, Alvarado’s scoring system, and acute appendicitis inflammatory scoring system. The parameters included in these systems are mainly based on patients’ physical examination results, signs and symptoms, and laboratory tests that are somehow different from the aforementioned scoring systems^25–27^. Selected features that have been applied in the current survey are based on these scoring systems. However, the included features were collected in the form of a survey. After confirming its validity and reliability, the form was distributed among 17 surgeons at three specialized hospitals: Imam Hossein, Taleghani, and Shahid Modarres. After collecting the survey forms, statistical calculations were performed on them to determine the priority of diagnostic features for the training process of artificial neural networks. Based on this, Cronbach's alpha coefficient of the above survey form was calculated to be 0.755, and since the obtained coefficient was greater than 0.7, it could be acknowledged that the survey form had good reliability^28^.

By using a collection form that included 16 features, the data of all 181 patients referred to the emergency department of Modares Hospital with abdominal pain and who had undergone appendectomy were collected during 2019 as a research database. The obtained data were collected in Excel file format by Excel 2016 software.

The required statistical analysis and tests among the characteristics has been published in our previous study^29^. The Student t-test or Mann–Whitney U-test was used for continuous variables, and the Chi-square test or Fisher's exact test, which are appropriate for categorical data, was used to assess univariate association across clinical or laboratory variables. The threshold of statistical significance was established as a two-tailed P < 0.05.

### Design, train and compare MLP and SVM

In this phase, an MLP and an SVM were used to classify the data, and the performance of each of these two systems in data classification was evaluated and compared for a number of different features. For this purpose, MATLAB software was used to design and train the perceptron neural network and the support vector machine system. During the training process, the K-fold cross-validation method was used to ensure the correct and complete training of the network and to evaluate the functional accuracy of the classification^30^. Some parts of predication results of various techniques were published in our previous work^29^.

Then, in the first case, for 11 to 16 diagnostic features, MLP and SVM were trained, and the results were compared. In another case, both algorithms, with diagnostic features other than laboratory parameters, were trained and evaluated to measure the impact of these parameters on the diagnostic process. The importance of this work is that if the impact of these parameters on the diagnostic process is low due to the time-consuming process of these tests, it is better to leave the diagnostic cycle. After training the techniques and comparing their diagnostic accuracy, we observe that laboratory parameters, especially leukocytosis, are inevitable in the diagnostic process. Three criteria—accuracy, specificity, and sensitivity—have been used to evaluate the performance of the classification system. Formulas (1–3)^31^ were employed to calculate these indices.

1 $$Accuracy=\frac{TP+TN}{TP+FN+TN+FP}\times 100$$

2 $$Specificity=\frac{TN}{TN+FP}\times 100$$

3 $$Sensitivity=\frac{TP}{TP+FN}\times 100$$

In order to evaluate the techniques in data classification, some data are used as training data and some as test data. In the evaluation of neural networks, typically, 10 to 20% of the data is used for network testing and the rest for network training. The K-fold cross-validation method has been used to evaluate the accuracy of classification systems. In this method, first, all data is randomly divided into K equal parts. The evaluation is done in K stage. In each step, the k-1 part of the data is used to train the network, and the remaining part is used to test its performance. Each part used for the experiment is different from the previous steps. Finally, the average accuracy, specificity, and sensitivity of different stages are reported as the system's accuracy, specificity, and final sensitivity.

### Interface design of CDSS

Later on, two main tools were used to construct and design the user interface in this study. The first tool that had to be installed on the system for Java programming was the "Java Development Tool." The next tool used in this study was the development environment of the NetBenz complex, which is one of the most complete Java programming environments.

### Evaluation of designed CDSS

Two stages of evaluation were performed on the designed system. The program was initially delivered to the academics of the medical informatics department at Tehran University of Medical Sciences for technical evaluation in focus groups, and their suggestions were taken into account to alter the system in phases.

**Figure 1:**
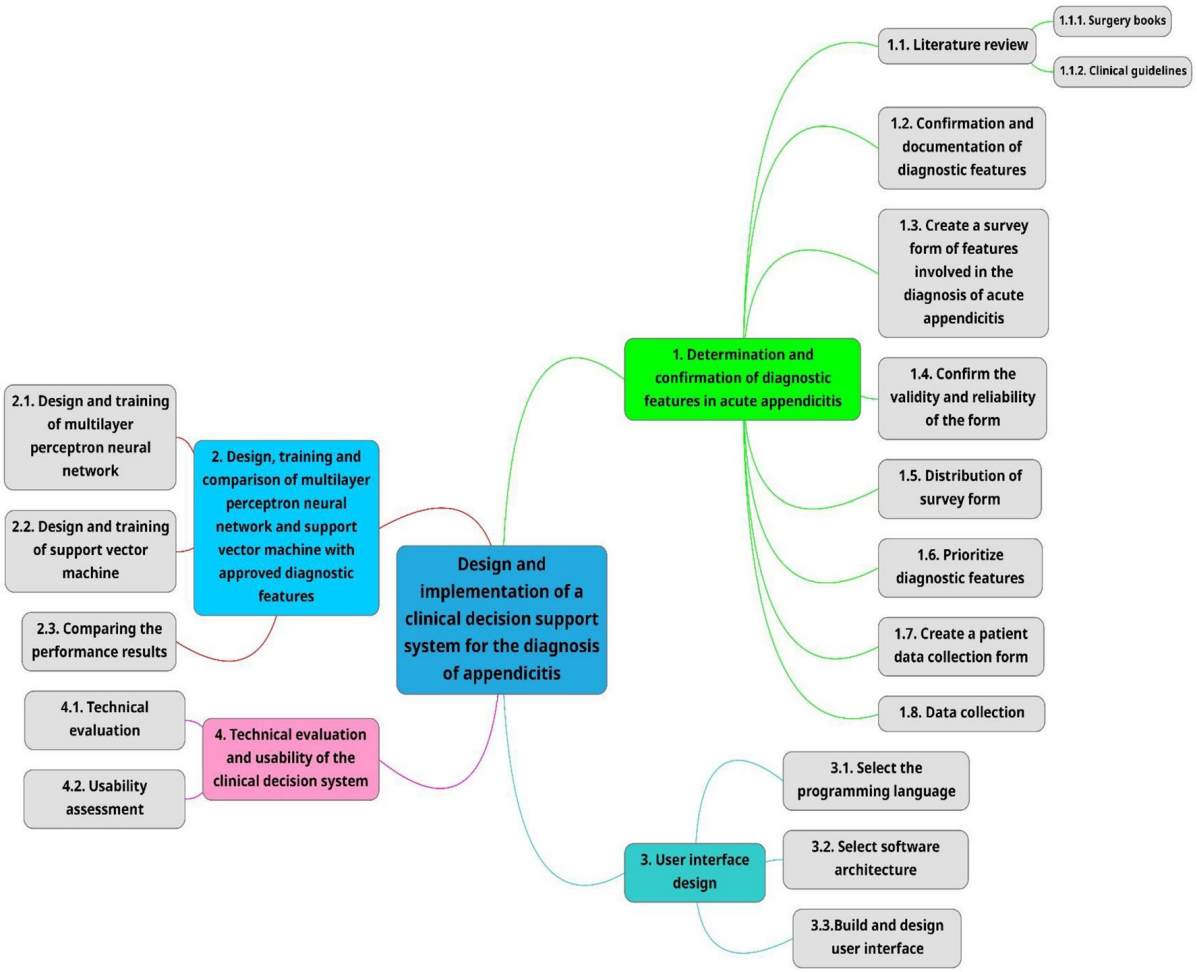
Mind map of applied methodology.

The primary goal of the system's creation, diagnostic accuracy, was assessed in order to gauge its utility. Retrospective usability testing was done utilizing pathology data from appendectomy patients as well as their patient information. The chosen SVM system was given a variety of information for this purpose, and its performance was assessed using the accuracy, sensitivity, and specificity criteria. The following mind map of the procedure is provided to help in understanding the actions conducted in this research (Fig. 1).

### Ethical aspects

All methodologies were conducted in adherence to pertinent rules and legislation. The approach employed in this study received approval from the Ethics committee of Tehran University of Medical Sciences. Verbal informed consent was obtained from all participants or their legal guardians for all stages of the study, and this procedure was approved by the ethics committee.

## Findings

### The extracted features for developing CDSS

After the collection of survey forms, information, and features were obtained and statistical indicators of mean, variance, and standard deviation were calculated for them. Thus, according to different professors, the features that had a higher diagnostic value for acute appendicitis were identified. Feature prioritization was used to reduce the features of the artificial neural network during the training, comparison, and network optimization process. The order of the mentioned features is given in Table 1.

**Table 1:** Order of selected features after the survey among 17 surgeons.

No	Scoring elements	Mean	Variance	Standard deviation	P-value
1	Migratory right iliac fossa pain	4.647	0.698	0.836	**0.002**
2	Right iliac fossa guarding	4.441	0.830	0.911	0.983
3	Right iliac fossa tenderness	4.294	0.560	0.748	**0.000**
4	Rebound tenderness right iliac fossa	4.058	0.996	0.998	0.164
5	Leukocytosis: 10.0–14.9 × 10^9^ cells/L ≥ 15.0 × 10^9^ cells/L < 10 × 10^9^ cells/L	4	1.058	1.028	**0.000**
6	Anorexia	3.882	1.280	1.131	0.728
7	Shift to the left of neutrophils	3.823	0.615	0.784	**0.000**
8	Rovsing’s sign	3.823	1.204	1.097	**0.011**
9	Right iliac fossa pain	3.647	1.993	1.411	**0.000**
10	Age: < 39.9 years > 40 years	3	0.941	0.970	0.960
11	Nausea or vomiting	2.941	0.643	0.802	**0.023**
12	Fever: ≥ 38.3 °C	2.882	0.927	0.962	0.272
13	Negative urinalysis	2.058	2.408	1.551	0.069
14	C-reactive protein concentration: 10–49 g/L ≥ 50 g/L	2.647	2.463	1.569	**0.000**
15	Gender: Male Female	1.058	1.820	1.349	**0.002**
16	Foreign nationality	0.823	2.027	1.423	**0.018**

Significant values are in bold.

Using the designed form, the information of 181 patients (126 men and 55 women) who had been referred to the emergency department of Modarres Hospital with abdominal pain and had undergone an appendectomy was collected in a database. First, 16 diagnostic features were measured and recorded in the emergency department. Then, after two weeks, the results of the pathology samples that had been sent from the operating room were added to the patient’s records. By performing calculations on the database of this study, we found that the opinions of surgeons were very close to the obtained statistics. According to the analysis, the average age in this study was 28 years old. Based on pathology reports, the accuracy of the correct diagnosis of acute appendicitis was 73.48%; hence, 101 of the 131 cases that had been correctly diagnosed were male and the remaining 30 were female, indicating that the disease was more than triple as common in men compared to women. Meanwhile, 26.51% of patients had normal appendicitis. The required statistical analysis and tests among the characteristics has been published in our previous study^29^. Sex, nationality, (migratory) right iliac fossa pain, nausea and vomiting, right iliac fossa tenderness, Rovsing's sign, leukocytosis, shift to the left of neutrophil, and CRP concentration were shown to differ significantly (P < 0.05) between the positive and negative pathology results.

### Development of MLP and SVM

For developing our intelligent CDSS, two techniques of MLP and SVM were used to classify the data. The performance of each of these two algorithms in data classification is then evaluated and compared for a number of different features.

#### Multilayer perceptron network

For designing an MLP, the number of neurons in the input layer, the number of hidden layers, and the number of neurons in each hidden layer must be determined. The number of input layer neurons is equal to the number of diagnostic features. The number of neurons in the output layer is considered equal to one. It is recommended that the number of hidden neurons be set to two-thirds of the size of the input layer, in addition to the size of the output layer. If the output of these neurons exceeds the threshold, assume the output to be one, meaning that these features belong to a person with appendicitis. If the output of these neurons is below the threshold, it indicates that the person is healthy.

**Figure 2:**
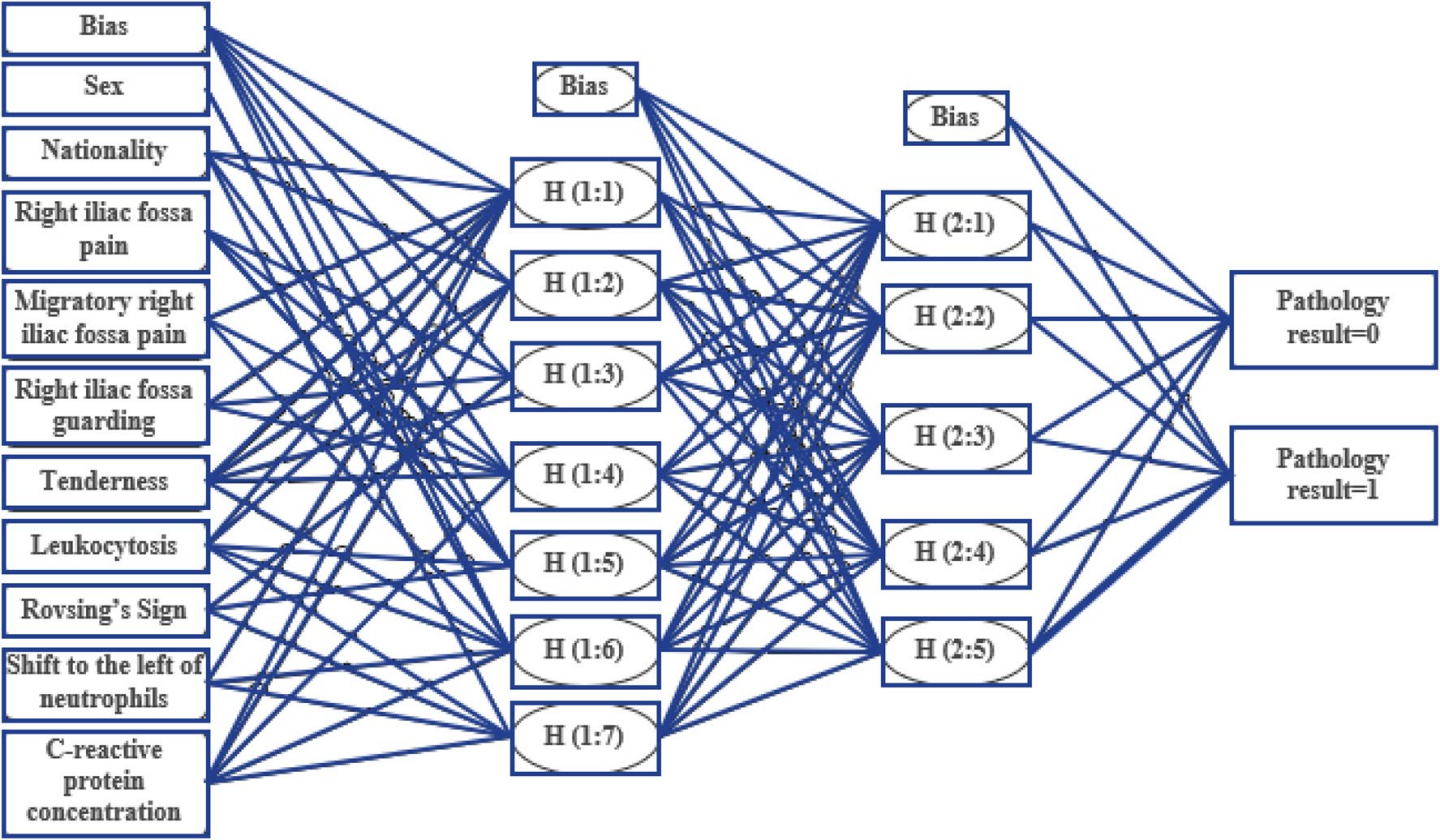
Structure of the MLP^29^.

During the training process of the MLP, the initial dimensions of the weights are randomly allocated. They then converge to a local or absolute minimum using learning algorithms. In most cases, the learning algorithm is trapped in a local ambush. The initial condition of weights is the starting point of the learning algorithm; therefore, it has a significant effect on the convergence of the algorithm to different local minima. In fact, by changing the initial conditions, the algorithm converges to different local minima, so the performance of an MLP with exactly the same structure changes for different initial conditions. In this research, to overcome this issue, each network has been weighted 100 times, i.e., starting with a random set of weights. Then, the average accuracy of network performance during these 100 experiments has been used as a criterion for comparing different structures. The network structure is given in the table for the applied features (Table 2). The optimal architecture of the developed MLP is illustrated in Fig. 2. Some details of MLP were provided with other methods^29^.

**Table 2:** MLP structure based on used features^32^.

Number of features	Input layer neurons	The first hidden layer neurons	The second hidden layer neurons	Output layer neurons
16	16	31	12	1
15	15	23	8	1
14	14	33	11	1
13	13	22	8	1
12	12	25	14	1
11	11	18	9	1

**Figure 3:**
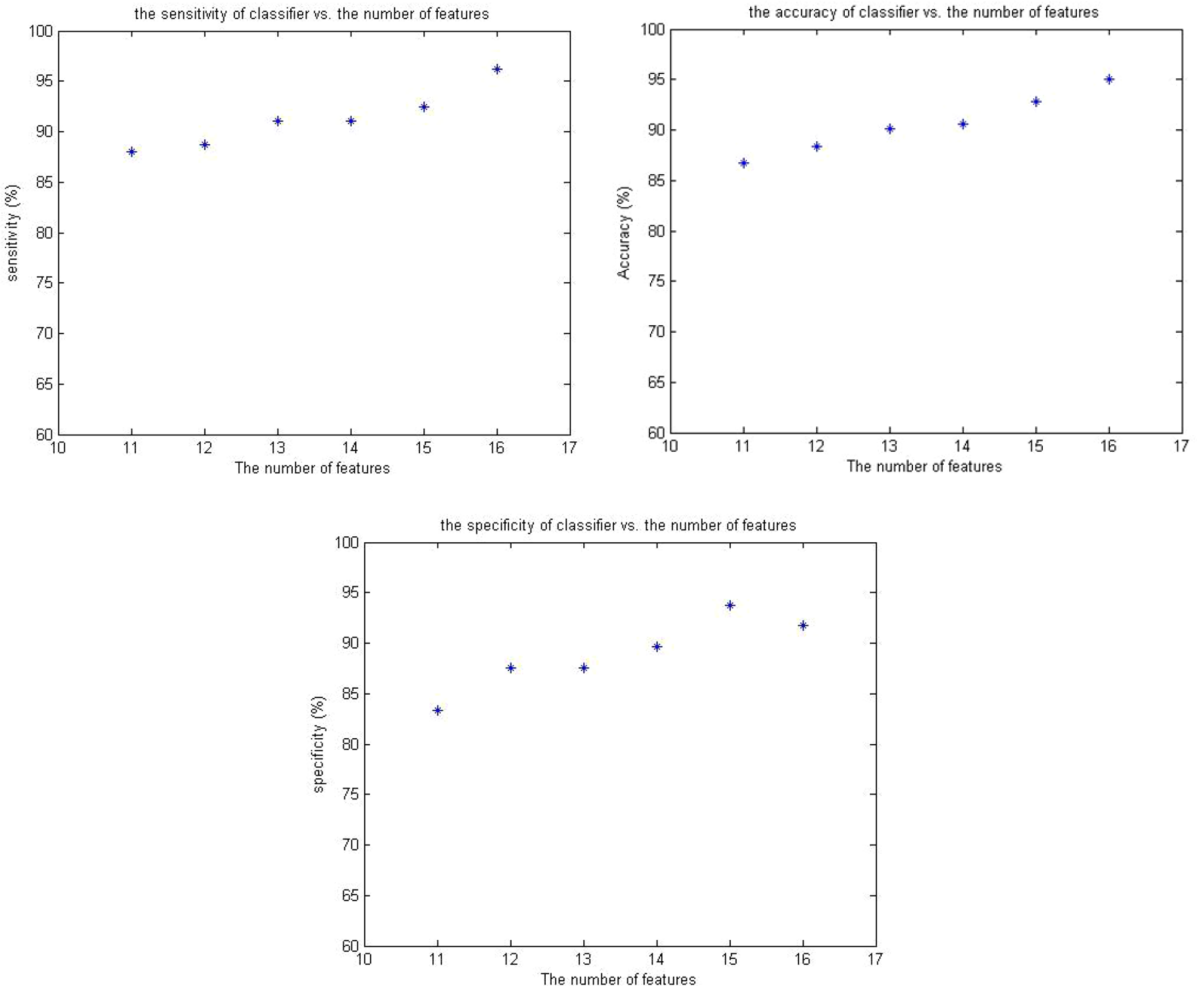
MLP performance in terms of number of features.

After ranking the features according to the surgeons, the MLP was optimized for 11 to 16 superior features and its performance was evaluated using the criteria of accuracy, sensitivity, and specificity. As shown, by limiting the number of employed features, the performance of MLP decreased; it can be inferred that utilizing more features is a way to enhance the performance of MLP. When all 16 extracted features were used in data classification, the mean accuracy, sensitivity, and specificity of the network were 78.5%, 82%, and 68.8%, respectively, which indicated a relatively good performance that could model the diagnosis of a general surgeon without the use of imaging techniques. Figure 3 shows the mean of accuracy, sensitivity, and specificity of the MLP network designed for a number of different features (mean of three indicators for tenfold-cross validation).

In the following, we examine the effect of laboratory features on the performance of the classification system. The features of leukocytosis, left shift in neutrophil count, CRP concentration, and negative urine analysis are determined by the laboratory. At this stage, by removing these four features and using other features, an MLP is designed and optimized to evaluate its performance without having these four features in hand. The mean accuracy, sensitivity, and specificity of MLP without laboratory features are 63.3%, 59.5%, and 66.7%, respectively.

#### Support vector machine

To design an SVM, the number of neurons in the input layer, the kernel function, and the number of neurons in the hidden layer must be determined. The number of input-layer neurons is equal to the number of diagnostic features. The number of neurons in the output layer is considered equal to one. If the output of these neurons exceeds the threshold, assume the output to be one, meaning that these features belong to a person with appendicitis. If the output of these neurons is below the threshold, it indicates that the person is healthy. In this research, the radial base function has been used as a nonlinear SVM kernel. To optimize this system, its radius value must be optimized. Based on our SVM model, we have established that the hinge loss function may be described as [0, 1 − yf(x)]. It should be noted that when the product of the predicted label (y) and the decision function (f(x)) is greater than or equal to 1, the hinge loss is equal to 0. Nevertheless, in cases where the value of yf(x) is less than 1, the hinge loss function experiences a substantial rise. The function yf(x) exhibits an increasing trend as the number of misclassified points, particularly those that are significantly erroneous, increases.

**Figure 4:**
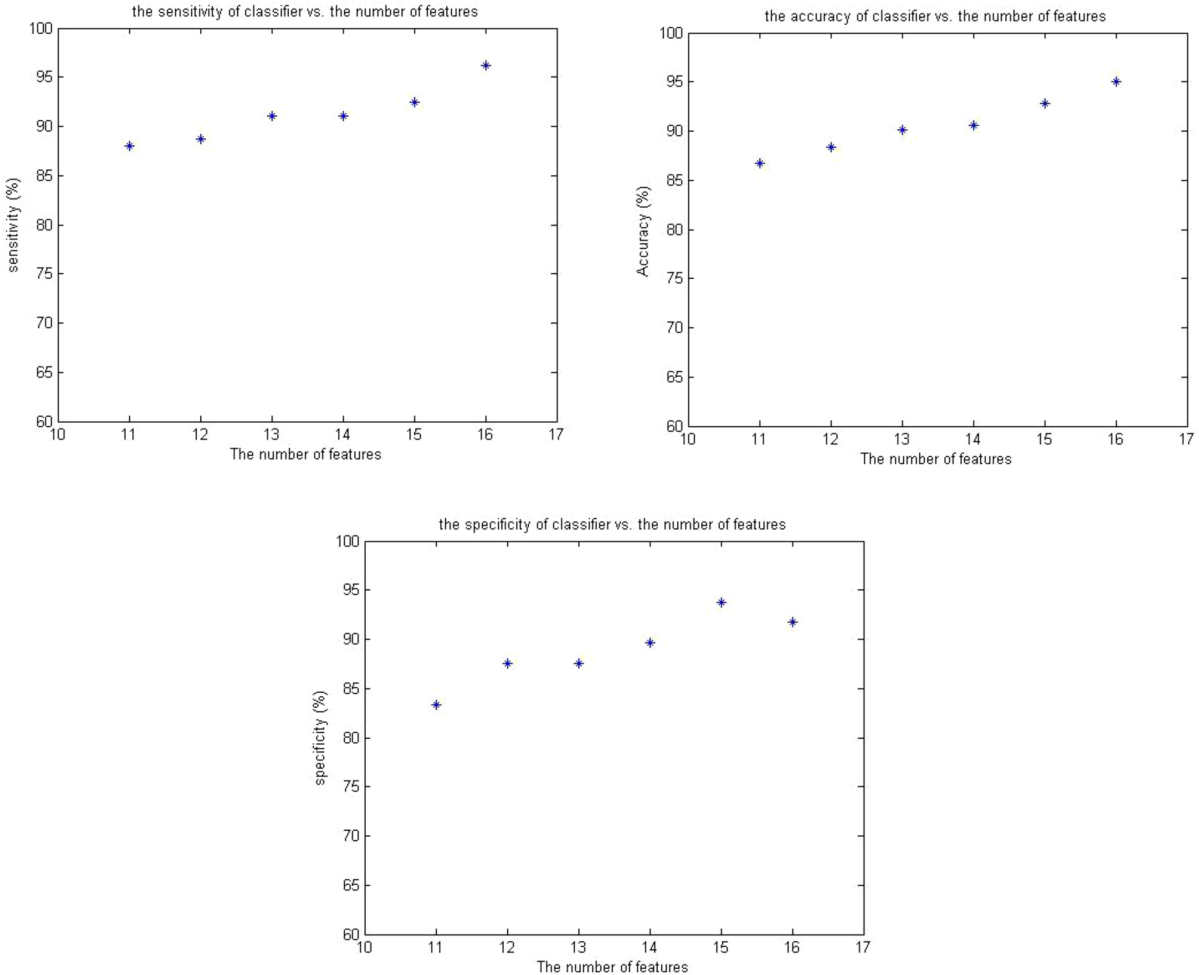
SVM performance in terms of number of features.

Then, the SVM was optimized for 11 to 16 superior features, and its performance was evaluated using the criteria of accuracy, sensitivity, and specificity. As it turns out, when all 16 extracted features were used in data classification, the mean accuracy, sensitivity, and specificity of the network were 95%, 96.2%, and 91.7%, respectively, which indicated very good performance. By reducing the number of features from 16 to 11, the accuracy, sensitivity, and specificity of the network were reduced to 86.7%, 88%, and 83.3%, respectively, which still indicated an acceptable performance that could easily model the diagnostic performance of a general surgeon without the use of imaging techniques. Hence, the following figures show the accuracy, sensitivity, and specificity of the SVM for a number of different features (Fig. 4, mean of three indicators for tenfold-cross validation).

In the following, we examine the effect of laboratory features on the performance of the classification system. The features of leukocytosis, left shift in neutrophil count, CRP concentration, and negative urine analysis are determined by the laboratory. At this stage, by removing these four features and using other features, an SVM is designed and optimized to evaluate its performance without having these four features in hand. However, the mean accuracy, sensitivity, and specificity of SVM without laboratory features are 66.9%, 69.9%, and 58.3%, respectively.

According to the results of training and evaluation of networks, it was found that the SVM with a basic radius kernel, in which 16 diagnostic features were used simultaneously, had the highest diagnostic power among the different networks and models tested. The network was able to correctly diagnose 128 of 133 patients with acute appendicitis and 44 of 48 patients with a normal appendix. Table 3 shows the diagnostic performance of the SVM, the MLP, surgeons, and pathologists in the database. More details about the chosen features and results of the train/test phase were described in the previous paper by the research team^29^.

**Table 3:** Number of correct and incorrect diagnoses.

Type of diagnosis	Diagnosis of surgical specialists	Pathologist diagnosis	SVM diagnosis	MLP diagnosis
Acute appendicitis	181	133	128	109
Normal appendix	0	48	44	33
Diagnostic accuracy (%)	73.48	100	95	78.5

By referring to the previous table (Table 3), the sensitivity, accuracy, and diagnostic specificity of the mentioned SVM system were 91.7%, 96.2%, and 95%, respectively. Due to the fact that, the accuracy of preoperative diagnosis should be above 85%, the performance of the SVM system designed to diagnose acute appendicitis was at a desirable level and could significantly prevent unnecessary surgery and related consequences. The SVM system with 16 inputs was selected as the processing core of the decision support system.

### User interface of designed CDSS

Here, the design of user interface of the CDSS is described:

The system consists of two main parts. The most important part, which is called "intelligent decision support", uses the optimized SVM system processing core that receives 16 features, including clinical examinations, patient statements, and laboratory tests, as input and then, presents the inputs in the form of an X vector through the following formula:

Nonlinear SVM classification formula:

4 $$C=\sum \limits_{i=1}\left. {a}_{i }K \left({s }_{i}, x\right)+\mathrm{b}\right)$$

**Figure 5:**
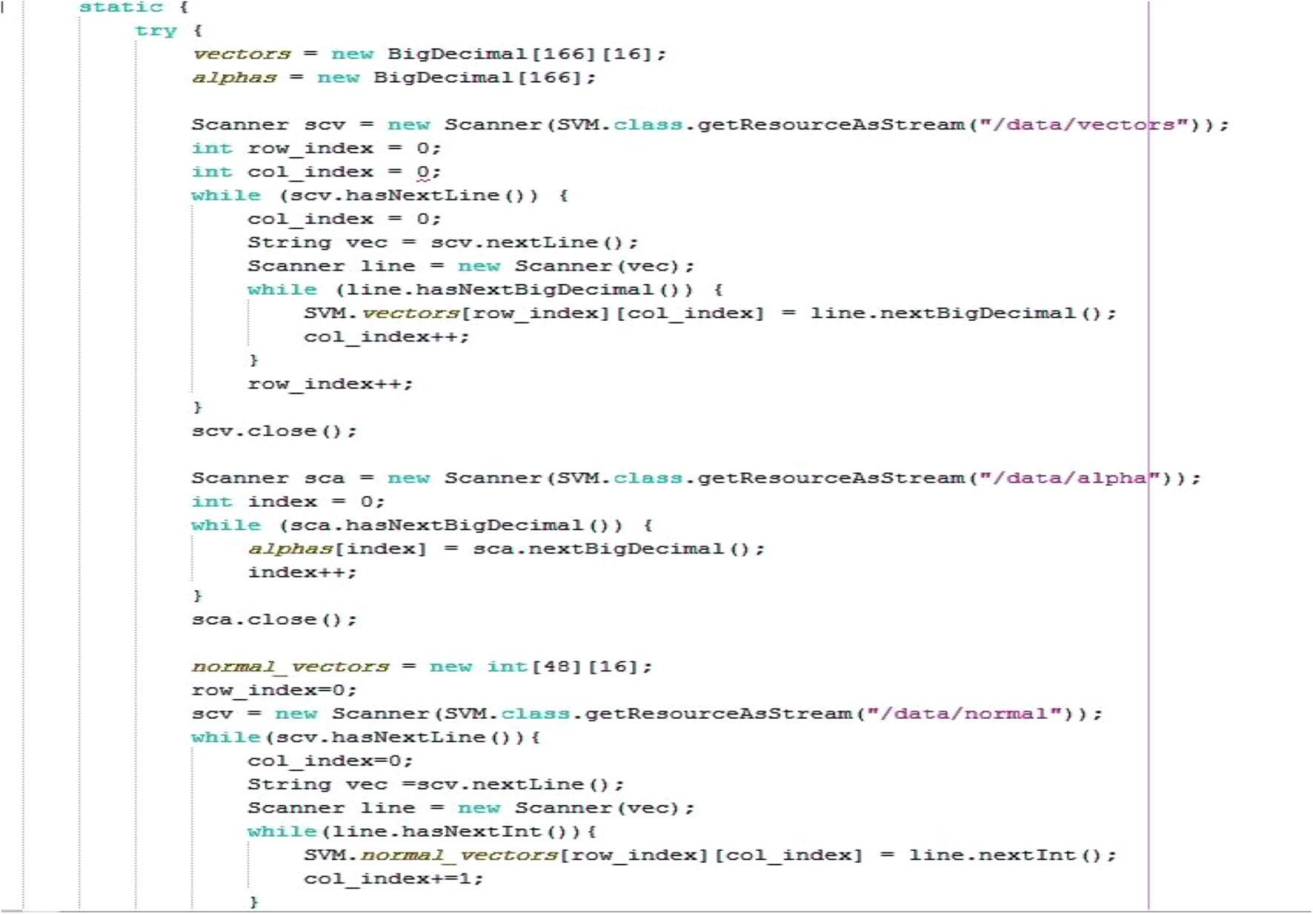
Part of the classification calculating code of the SVM in Java.

In this vector, s_i_ is the support vector, α_i_ is the weights vector, b is the bias (− 0.3090) and k is the kernel function. If c is greater than or equal to zero, then the patient will be classified in the first group (healthy), otherwise the patient is classified in the second group (patient). Figure 5 shows some of the code equivalent to the above formula in the Java programming language.

Another major part of the software called "optional scoring systems" provides the user with the three scoring systems of Alvarado, Rapasa, and inflammatory response of appendicitis, which, by selecting the inputs, provides the corresponding score and suggested instructions to the physician. Applying this extra scoring method will allow doctors to compare the primary outcomes of our main system, which is based on normality and appendicitis, with the outcomes of other international scoring systems. In addition, a section called "About Us", was provided to display information about software and research.

**Figure 6:**
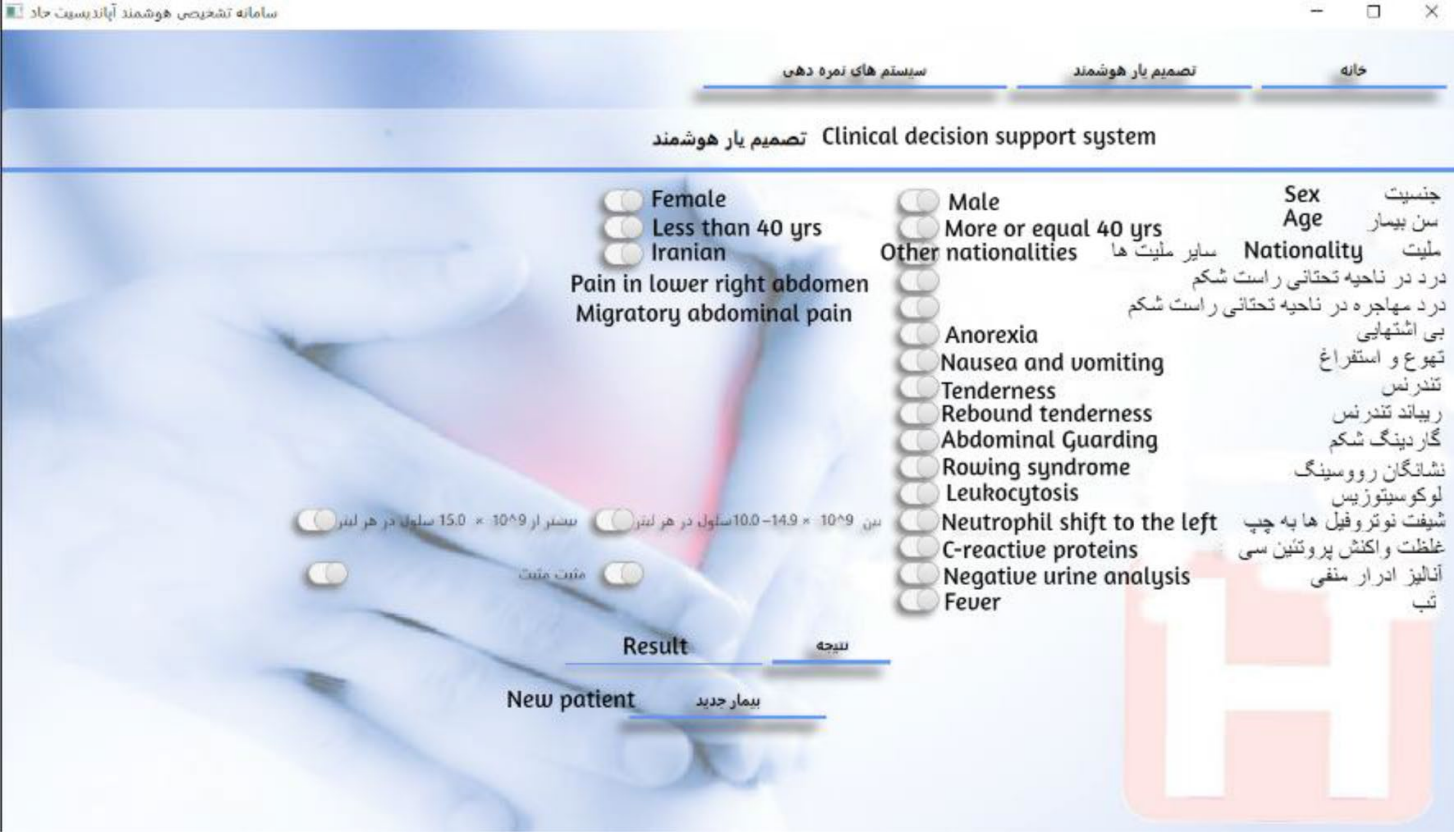
A view of main CDSS page.

In the "Intelligent Decision Support" section, the four features of leukocytosis, lower right abdominal guarding, tenderness, and radiating pain in the lower right abdomen were marked by a "star" with the opinion of the clinical consultant (Fig. 6). Selected features had the highest scores in the surgeons’ survey. Similarly, features with high diagnostic values would have a higher weight in the network training process. As a result, they affect the correct performance of the SVM system and its classification.

**Figure 7:**
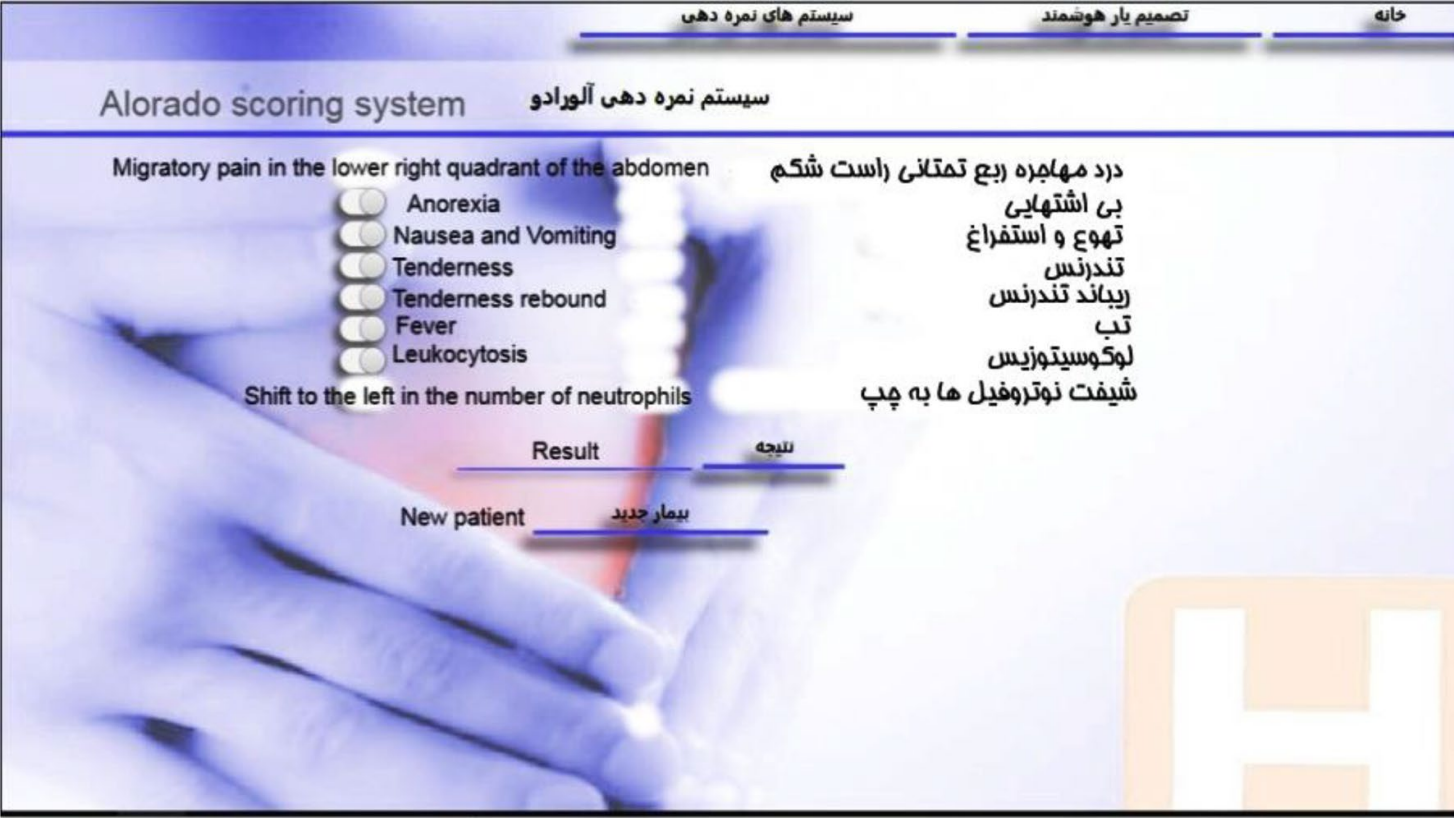
A view of the “Alvarado scoring system” page.

In the Alvarado scoring system (Fig. 7), by selecting the features, the related calculations are performed and the result is displayed in the form of a score, suggestion or related clinical probability.

**Figure 8:**
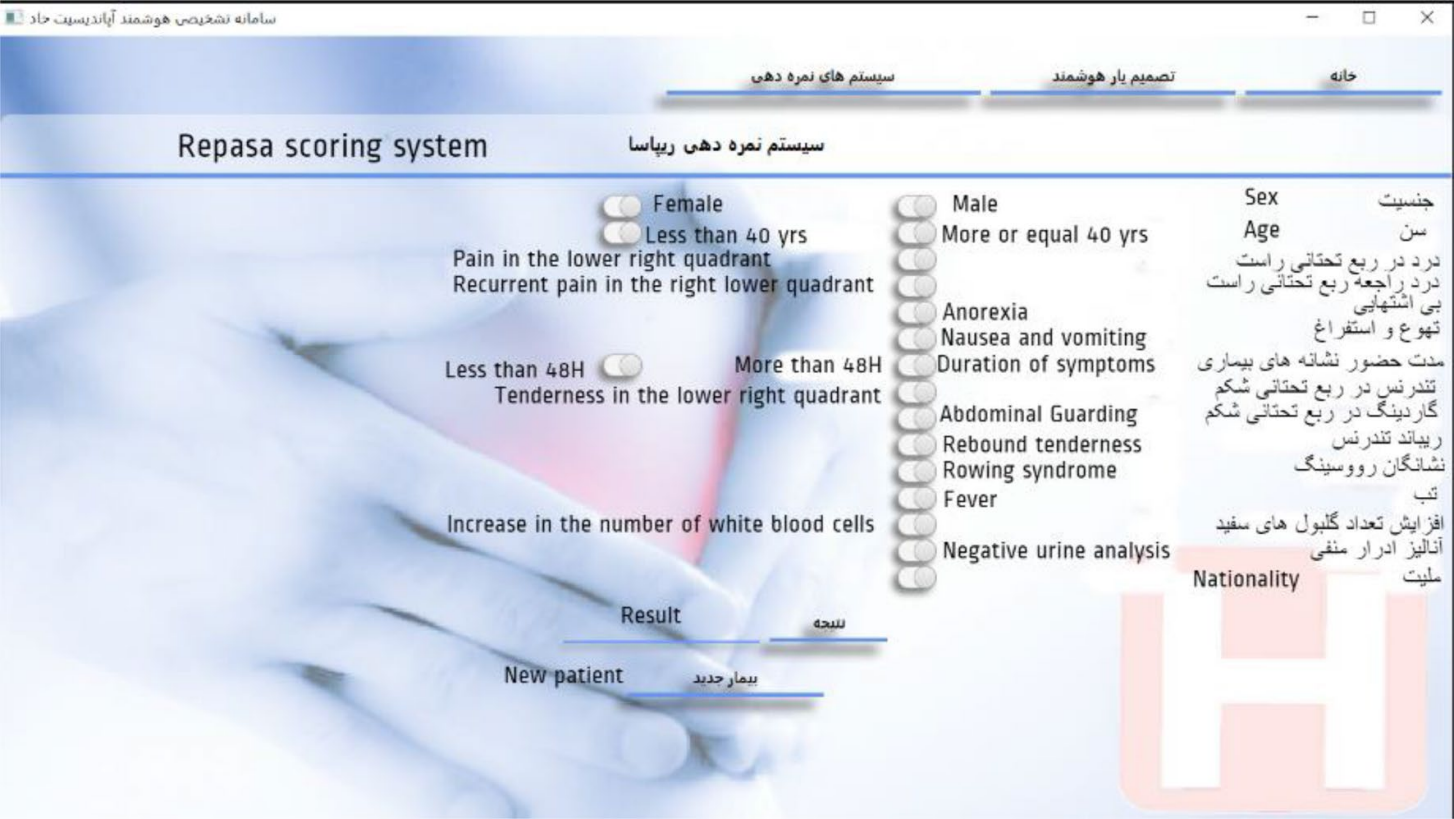
A view of the “Repasa scoring system” page.

In the decision support of the Repasa scoring system (Fig. 8), by selecting the features, the related calculations are performed and the result is displayed in the form of a score.

**Figure 9:**
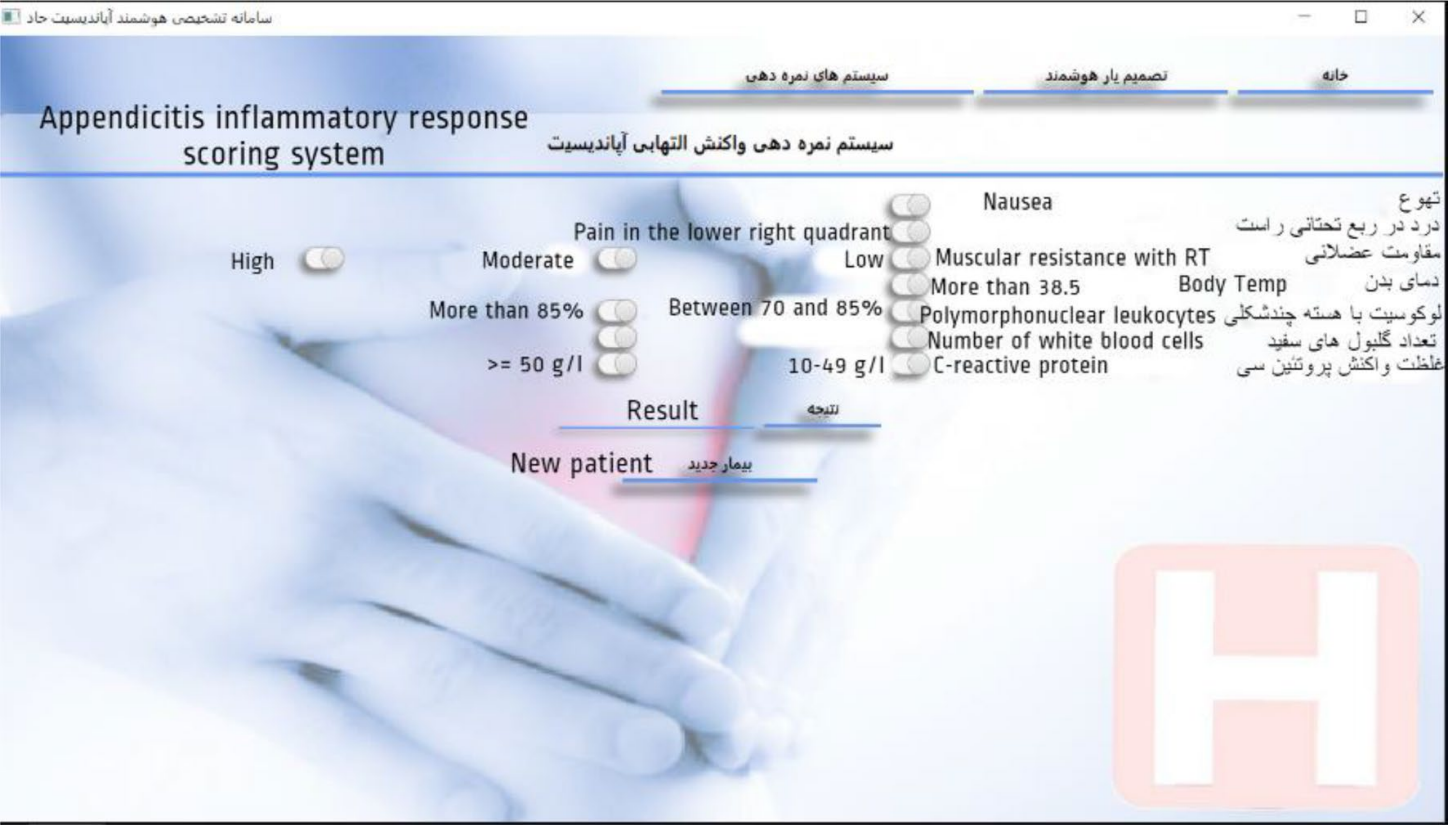
A view of the “appendicitis inflammatory response scoring system” page.

In the decision support of the acute appendicitis inflammatory response scoring system (Fig. 9), by selecting the features, the related calculations are performed and the result is displayed in the form of a score, suggestion or related clinical probability.

### Evaluation of the CDSS

The evaluation of the diagnostic system was performed in two stages. Initially, for its technical evaluation (in focus groups), the software was presented to the professors of the medical informatics department at Tehran University of Medical Sciences and their opinions were used to modify the system in several stages. Modifications included changes to the main and sub-classes, followed by changes to the coding and appearance of various parts of the program.

**Figure 10:**
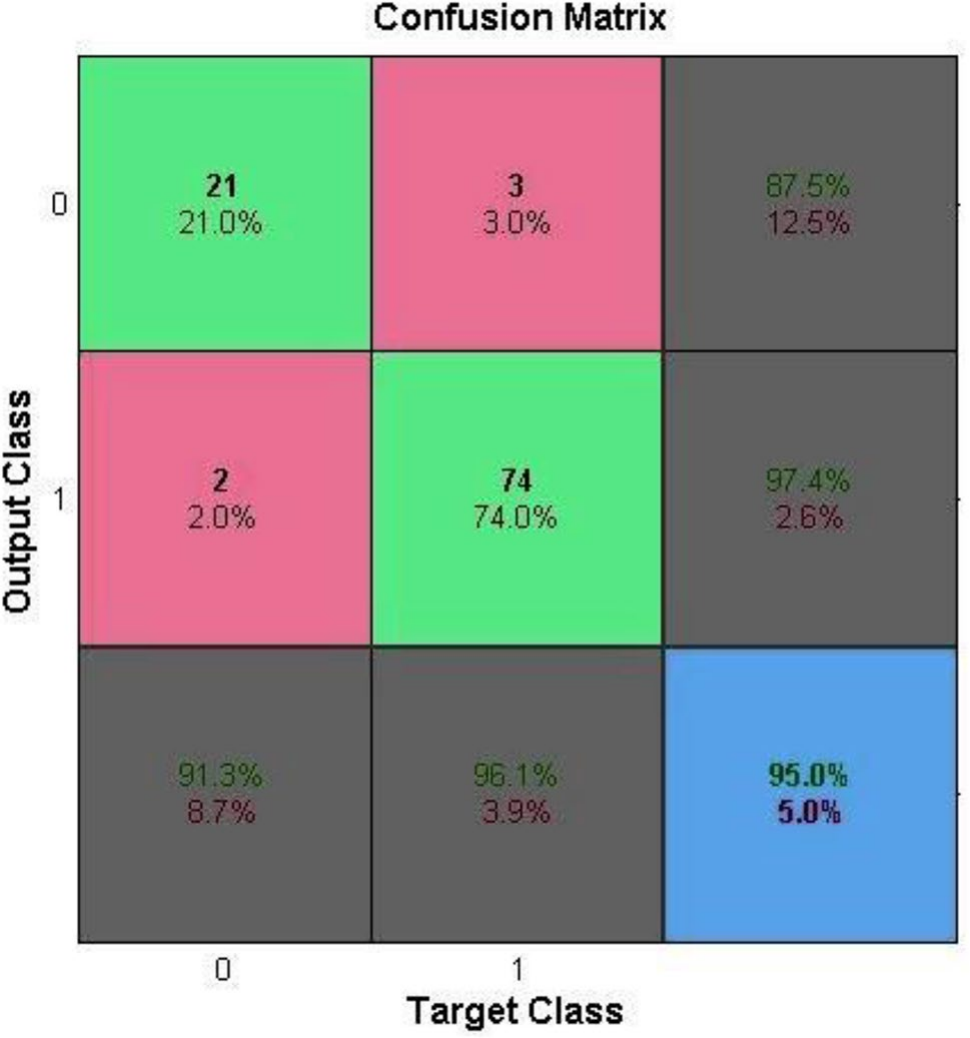
Results of the system usability assessment.

To evaluate the system's usability, the chief purpose of its creation, which was diagnostic accuracy, was evaluated. A retrospective usability assessment was performed utilizing the information of patients who had undergone appendectomy and whose pathological results had been determined. For this purpose, the information of one hundred patients was collected separately, chosen, and fed to the processing core of the CDSS (Fig. 10).

In Table 4, results of usability assessment of the system have been compared with the pathology results.

**Table 4:** Comparison of diagnostic performance of the processing core of decision support system and pathology results.

Comparison of the pathology and processing core diagnosis		Pathology diagnosis		Total
		Acute appendicitis	Normal appendix	
SVM diagnosis	Acute appendicitis	74	3	77
	Normal appendix	2	21	23
Total		76	24	100

According to these results, it is clear that the accuracy, sensitivity, and specificity of the evaluation undertaken were 95%, 96.1%, and 91.3%, respectively. The processing core of the decision support system, namely the SVM algorithm, was able to correctly classify 95 out of 100 items that were given to it. Considering the results obtained from this section, it can be acknowledged that the system designed to diagnose acute appendicitis, which is the main purpose of its construction, works very well.

## Discussion

The results obtained in this research can be explained in several sections. First, the necessary features in the diagnosis of acute appendicitis were discussed. In our study, after a literature review, the use of standard guidelines and consultation with physicians, the features were surveyed for prioritization in several stages among the surgical specialists of the three hospitals. In addition, it was found that pain in the lower right abdomen is not the only diagnostic feature that is of great importance, but also other features such as migratory pain in other areas of the abdomen are more important than it^33^.

Considering the high volume of patients admitted to the emergency department, especially in governmental medical centers in developing countries, the time-consuming nature of imaging methods, and the time limit for diagnosing acute appendicitis, it will be beneficial to use machine learning and artificial intelligence technologies to help diagnose appendicitis, according to the diagnostic characteristics of societies, which will speed up the process of diagnosing the disease, reduce the costs and duration of the patient's stay in the emergency department, and prevent a negative appendectomy^34^. A significant deficiency in specialist services and the corresponding infrastructure is observed in the majority of rural health centres situated in developing countries. Therefore, the implementation of such a tool could prove to be beneficial in the context of Remote Health Care (RHC), as it would aid general physicians in effectively screening acute appendicitis individuals and promptly referring them to specialised healthcare facilities^35^.

In the present study, two classification techniques, specifically MLP with the post-propagation algorithm employing maximum gradient reduction with momentum, and a nonlinear SVM system utilising the base radius kernel, were employed. After conducting a comparison between the optimisation processes of the MLP network and the SVM system, it can be inferred that the SVM optimisation system exhibits superior speed and reliability. Research has demonstrated that SVMs may exhibit longer execution times due to the inclusion of computationally intensive operations, such as the utilisation of kernel functions to transform data into higher-dimensional spaces. However, it consistently demonstrates a high level of accuracy in its predictive capabilities.

In 2014, Sung Yun and colleagues conducted a study titled "Application of an artificial intelligence method for diagnosing acute appendicitis: the support vector machine" in Korea. The study aimed to compare the effectiveness of three diagnostic methods, namely the Alvarado scoring system, MLP networks, and SVM, in diagnosing acute appendicitis. The diagnostic accuracy of the Alvarado scoring system, MLP networks, and SVM was determined to be 54.87%, 92.89%, and 99.61%, respectively. Based on our investigation, the findings demonstrate the significant diagnostic efficacy of SVM systems in the identification of medical conditions, particularly acute appendicitis, in comparison to MLP networks. This distinction can potentially enhance both the precision and efficiency of illness diagnosis^23^.

A mathematical model was constructed with Pain-Only-Parameters (POP) sourced from existing literature in order to conduct screening for Acute Appendicitis (AA). Weights have been allocated to each point of pain (POP) in order to construct a training data matrix consisting of 51 observations. These weights are then utilised to compute the cumulative effect, also known as the Pain Confidence Score (PCS). According to the Patient Classification System (PCS), a cohort of actual patients is categorised as either case of AA or non-appendicitis (NA), yielding favorable outcomes that align with our own findings (with a sensitivity of 85%, specificity of 75%, precision of 77%, and accuracy of 80%)^35^.

In a previous study, neural networks were employed to diagnose liver ulcers, similar to the current investigation. The findings derived from this study demonstrate that the SVM system exhibits superior performance, achieving a diagnostic accuracy of 98%, in contrast to the artificial neural network which achieved a diagnostic accuracy of 96%. These results suggest that the SVM method outperforms the post-propagation method in the diagnosis of liver ulcer disease^36^. Based on the findings derived from our study and references to relevant literature, it can be inferred that the implementation of an optimised SVM system holds the potential to expedite and enhance disease detection by medical practitioners, thereby substantially mitigating the adverse consequences associated with misdiagnosis.

In a study by Tenorio and colleagues in Brazil^37^, artificial intelligence techniques were used to create a CDSS for the diagnosis of celiac disease. In this study, five artificial intelligence techniques, including decision trees, Bayesian networks, nearest neighbor algorithms, SVM, and artificial neural networks, were trained and compared. Among the mentioned methods, the most accurate diagnosis with 80% accuracy, 78% sensitivity, and 80% specificity was related to the Bayesian classifier, which was used as the processing core of the web-based CDSS. To evaluate the usability of his system, the research team retrospectively provided a database of 38 patients to the clinical decision support system and compared the results with physicians' diagnoses and existing standards for this diagnosis. The comparison showed that the diagnostic results of the CDSS (kappa coefficient 0.68%) and physicians' diagnoses (kappa coefficient 0.64%) are both very close to the diagnosis that can be given according to international standards. In this study, 178 samples were used as a database for training artificial intelligence techniques, but in the evaluation and testing of the decision-making system, only 38 samples were used as a test database, which can reduce the reliability of evaluation results. In our study, to evaluate the system's usability, 100 samples were collected and chosen. The intelligent diagnostic system was designed in the Java programming language. This design allows the program to run on any operating system without the need for Internet access. This makes the system easier to access and use. In another study, three machine learning algorithms were used to predict the survival rate after kidney transplantation. Of the three models, the C5.0 algorithm was the best model with high reliability, which proved its power in predicting the survival rate. To identify the effective factors in predicting transplant survival, information needs analysis was performed through a researcher-made questionnaire ^38^.

In contrast with our study, in 2009, Mr. Putdokhe and Karule used the MLP network as the processing core of their decision support system for the analysis of liver ultrasound and imaging and diagnosis of liver disease. In this study, which was performed with an image processing technique, the performance of the three methods of MLP network, basal radius function network, and SVM system was compared. Finally, the MLP network with the highest diagnostic accuracy was used as the processing core of the decision support system for liver imaging to help analyze and diagnose liver diseases ^39^. Using image processing methods as the processing core of an application requires strong hardware to install the software. This can be problematic due to the high cost of hardware^40^. This is while, our intelligent system has an optimized processing core that achieves the result with a few calculations, and the hardware and software requirements of this system are minimal.

In a parallel study, an examination was conducted on demographic and laboratory data, employing several machine learning techniques, to ascertain the likelihood of surgical intervention for pediatric patients presenting with suspected acute appendicitis. The technique of gradient augmentation yielded the highest level of accuracy, reaching 95%, which is comparable to the accuracy achieved by our created SVM^41^. Furthermore, in a separate investigation, the diagnosis of appendicitis in children was made utilising demographic data prior to the performance of laboratory procedures. Furthermore, a distinction was made between cases of complicated and uncomplicated appendicitis. In this study, the decision tree model achieved AUROCs of 0.94 and 0.79 for the prediction of appendicitis and uncomplicated appendicitis, respectively^42^. The appendicitis scoring system was used for a dataset of pediatric patients who presented with abdominal pain to predict the diagnosis of perforated appendicitis^24^. The Heidelberg score was modified and a data-driven score was created using decision trees and random forests, yielding AUROCs of 0.92 and 0.86, respectively, for the diagnosis of appendicitis and both 0.71 for perforation^43^.

In a study, Lorenzo-Zúñiga et al. developed an intelligent clinical decision support system for the automated detection of colorectal gastrointestinal tract conditions using endoscopic films. The system demonstrated a high diagnostic accuracy, successfully identifying 94% of adenomas in the digestive system, even when presented with low-quality endoscopic images. The development and utilisation of an intelligent clinical decision support system were undertaken specifically for the purpose of telemedicine applications^44^. In line with our work, this system has very good diagnostic accuracy, considering the method of its implementation, which is the processing of endoscopic film frames. The use of telemedicine architecture has increased its accessibility and usability, which is very beneficial. However, it is difficult to prepare the necessary substrates for using the system for telemedicine^45^. Meanwhile in our study, the designed intelligent diagnostic system runs in any operating system with a Java interface and does not require any special substrates.

Valente et al. in their research showed that reporting the amount of water consumed is one of the most important factors in preventing the formation of urinary tract stones. The ability to record and report water consumption by smartphone-based applications helps to send appropriate reports to urologists who then prescribe medical prescriptions, and this ultimately improves the quality of life of patients^46^. In this program, it is also possible to record the water consumption and pH of urine, which can be used to better view the patient's condition using graphical reporting.

In a study conducted in 2012, Anooj et al.^47^ employed fuzzy weight rules in the development of a CDSS aimed at assessing the likelihood of cardiac disease. The present work involved the initial extraction of features from the UCI repository through the utilisation of data mining techniques. The features that were acquired were afterwards utilised through the implementation of fuzzy weighted rules. The CDSS was developed based on the rules that were acquired. Subsequently, a comparative analysis was conducted between the obtained results and those derived from an artificial neural network that underwent training using the identical database. The assessment of the developed system was conducted by employing the k-fold cross-validation technique on three pre-existing databases. The findings of this study indicate that the artificial neural network exhibited superior performance compared to the designed fuzzy system. The research database referred to in this context is a pre-existing database that encompasses a range of countries and is accessible over the Internet. Based on the findings of this study and considering the variable nature of cardiac diseases under different circumstances, it is advisable to establish region-specific databases in order to enhance the reliability of the results^48,49^. However, in our study, a database with optimized features for regional conditions was designed and created to train the artificial neural network and the SVM.

### Limitations

One of the shortcomings of this study is that the results are based on data from only one hospital. However, the other limitation is that the surveying to identify the necessary parameters was conducted only by 17 surgeons from three hospitals, which might diminish the generalizability of the findings of the first phase of the research. Adding more detection features can increase the value of the work; yet, we had limited time and budget in this study, and because of these challenges, we could not include more features in machine learning-based techniques. In future studies, this case can be taken into consideration and more studies can be done to increase the accuracy of the diagnosis. Finally, this CDSS was not evaluated in a real clinical setting.

## Conclusion

The developed intelligent diagnostic system produced the desired results and efficiency. According to the results of the training and evaluation of networks, it was found that SVM with a basic radius kernel in which 16 simultaneous diagnostic features were used had the highest diagnostic power among the networks and different modes tested. This system in medical centers can be used for timely diagnosis, preventing negative appendectomy, reducing patient hospitalization time and treatment costs, and improving the patient referral system. Thus, this system helps physicians make a faster, more accurate, and more emergency diagnosis of the disease and significantly reduces the use of imaging techniques, complications of late diagnosis, unnecessary appendectomy, length of hospital stay, and treatment costs. It is suggested that more research center data be used in future studies, that the results be compared, and that the system be evaluated in an empirical clinical setting.

## Data Availability

All data generated or analyzed during this study are included in this published article**.**
